# Data on entrepreneurship education and entrepreneurial performance of aspiring entrepreneurs in selected Nigerian universities

**DOI:** 10.1016/j.dib.2018.07.044

**Published:** 2018-07-27

**Authors:** Mercy Ejovwokeoghene Ogbari, Maxwell Ayodele Olokundun, John Uzuegbunam, David Taiwo Isiavwe, Julie Enamen Ilogho, James Nwonye Obi, Chinonye Love Moses

**Affiliations:** a Department of Business Management, College of Business and Social Sciences, Covenant University, Ota, Nigeria; b Department of Accounting Igbenedion University Okada, Edo State, Nigeria; c Centre for Learning Resources, Covenant University, Ota, Nigeria

**Keywords:** Entrepreneurship education, Entrepreneurial performance, Aspiring entrepreneurs, University, Nigeria

## Abstract

Entrepreneurs are individuals who have a constant feeling of desperation that is from time to time seen in organizations. While the number of entrepreneurship education programmes are increasing, their impact is under-researched and studies paint an unclear picture of the impact of entrepreneurship education. This present study presents data on the extent to which university entrepreneurship education programmes stimulate the entrepreneurial performance of aspiring entrepreneurs in Nigeria. Data was collected using a descriptive cross-sectional quantitative survey conducted among university students (*N* = 540) of selected institutions in Nigeria. Regression Analysis was used in confirming the hypotheses proposed in the study using the Statistical Package for Social Sciences (SPSS) version 22. University entrepreneurship education is confirmed to be a major source of inspirational triggers that positively impact on entrepreneurial performance of aspiring entrepreneurs in the selected universities in Nigeria. The field data set is made widely accessible to allow for critical inquiry.

**Specification Table**

**Table:** 

**Subject area**	Business, Management
**More Specific Subject Area:**	Business and Entrepreneurship education
**Type of Data**	Table
**How Data was Acquired**	Researcher-made questionnaire analysis
**Data format**	Raw, analyzed, Inferential statistical data
**Experimental Factors**	Sample consisted of university students in Nigeria. The researcher-made questionnaire which contained data on entrepreneurship education and entrepreneurial performance of aspiring entrepreneurs were completed.
**Experimental features**	Teaching entrepreneurship in the university context is a major determinant of entrepreneurial performance of aspiring entrepreneurs in Nigeria.
**Data source location**	South west Nigeria
**Data Accessibility**	Data is included in this article

**Value of Data**

• The data presented described the effect of entrepreneurship education on entrepreneurial performance of aspiring entrepreneurs in Nigeria. However, to afford better generalizability, we call on other researchers to replicate our survey in different settings and context.
• Future research studies that rely on a different context as sources may help explicate whether the data described here are informant-sensitive.
• Future studies might consider expanding their investigation to include other potential factors of entrepreneurial performance of aspiring entrepreneurs aside from entrepreneurship education.

## 1 Data

The data for this research was collected from university students in three Nigerian institutions. A total of six hundred (540) copies of questionnaire were distributed and three hundred and eighty-two (382) copies were returned representing seventy one percent (71%) response rate. The study adopted descriptive cross-sectional survey research design in which the research questionnaire was administered to respondents based on purposive, stratified and simple random sampling techniques. Table 1 below shows the allocation of copies of the questionnaire based on proportionate ratio (Table 2).

**Table 1:** Total questionnaire distributed.

**Copies of questionnaires**	**Frequency**	**Valid percent**
Number of copies of questionnaire returned	382	71%
Number of copies of questionnaire not returned	158	29%
Total	540	100

**Table 2:** Allocation of copies of questionnaire.

**University**	**Estimated Response**	**Actual Response**
Covenant University	195	161
Landmark University	130	108
University of Lagos	215	113
	540	382

### 1.1 Test of Hypotheses

H01 There is no significant interdependence between entrepreneurial programmes and students’ entrepreneurial ventures.

The table above is a model summary. It shows how much of the variance in the dependent variable (entrepreneurial ventures) is explained by the model (university entrepreneurship programmes). The *R* square value is 0.76, expressed by a percentage; this means that our model (entrepreneurship programmes affect entrepreneurial performance) explains 7.6% of the variance in entrepreneurial ventures. The adjusted *R* square shows 0.72 while the standard error estimates designate 0.91087 which signifies the error term that was not captured in the model (Table 3, Table 4, Table 5, Table 6, Table 7, Table 8).

**Table 3:** Model summary. Source: Field Survey 2016.

Model	*R*	*R* Square	Adjusted *R* Square	Std. Error of the Estimate	Change Statistics				
					*R* Square Change	*F* Change	df1	df2	Sig. *F* Change
1	.276^a^	.076	.072	.91087	.076	15.672	2	379	.000

a Predictors: (Constant), FD, FC.

**Table 4:** ANOVA^a^. Source: Field Survey 2016.

Model		Sum of Squares	Df	Mean Square	*F*	Sig.
1	Regression	26.006	2	13.003	15.672	.000^b^
	Residual	314.447	379	.830		
	Total	340.453	381			

a Dependent Variable: FB.

b Predictors: (Constant), FD, FC.

**Table 5:** Coefficients^a^. Source: Field Survey 2016.

Model		Unstandardized Coefficients		Standardized Coefficients		*T*	Sig.
		*B*	Std. Error		Beta		
1	(Constant)	2.253	.182			12.411	.000
	FC	.252	.046		.282	5.501	.000
	FD	−.110	.045		−.126	−2.461	.014

a Dependent Variable: FB.

**Table 6:** Model summary. *Source*: Field Survey 2016.

Model	R	R Square	Adjusted R Square	Std. Error of the Estimate
1	.321^a^	.103	.098	1.05616

a Predictors: (Constant), IC, IA.

**Table 7:** ANOVA^a^. *Source*: Field Survey 2016.

Model		Sum of Squares	Df	Mean Square	*F*	Sig.
1	Regression	48.570	2	24.285	21.771	.000^b^
	Residual	422.763	379	1.115		
	Total	471.332	381			

a Dependent Variable: IE.

b Predictors: (Constant), IC, IA.

**Table 8:** Coefficients^a^. *Source*: Field Survey 2016.

Model	Unstandardized Coefficients		Standardized Coefficients		*t*	Sig.
	B	Std. Error	Beta			
1	(Constant)	4.540	.218		20.786	.000
	IA	−.027	.053	−.025	−.507	.613
	IC	−.314	.048	−.325	−6.563	.000

a Dependent Variable: IE.

The table above shows the assessment of the statistical significance of the result. The ANOVA table tests the null hypothesis to determine if it is statistically significant. From the results, the model in this table is statistically significant (sig = .000) in which the *F*- value is equal to 15.672 and the *p* value less than 0.05.

The table above demonstrates which of the variables included in the model contributed to the prediction of the dependent variable. The study is interested in comparing the contribution of each independent variable; therefore, beta values are used for the comparison. In this table, the largest beta coefficient is .282, which relates to level of interdependence between entrepreneurial ventures and university entrepreneurship education programmes.

H02 University entrepreneurial education has no significant effect on the student product development.

The table above is a model summary. It shows how much of the variance in the dependent variable (student product development) is explained by the model (entrepreneurship education). The *R* square value is .103, expressed by a percentage; this means that our model (entrepreneurship education has a significant effect on student product development) explains 32.1% of the variance in student product development. The adjusted *R* square shows 0.98 while the standard error estimates designate 1.05616 which signifies the error term that was not captured in the model.

The table above shows the assessment of the statistical significance of the result. The ANOVA table tests the null hypothesis to determine if it is statistically significant. From the results, the model in this table is statistically significant (sig = .000) in which the *F*- value is equal to 21.771; when the significance is less than 0.05.

The table above demonstrates which of the variables included in the model contributed to the prediction of the dependent variable. The study is interested in comparing the contribution of each independent variable; therefore, beta values are used for the comparison.

## 2 Experimental design, materials and methods

Data was gathered from students in three selected Nigerian universities (Covenant University, Landmark University and University of Lagos) with the help of a customized questionnaire crafted in alignment with the studies of [1], [2], [3], [4], [5]. The collected information was coded and inputted in SPSS variant 22. Data analysis was done; utilizing SPSS-22. Data was analysed using inferential tests specifically; regression analysis. Survey research design was adopted for this study where data was collected from a sample size of five hundred and forty students from the three universities to determine the strategic impact of university entrepreneurship education on entrepreneurial performance of aspiring entrepreneurs. The questionnaire was self-administered to the respondents who willingly filled the research questionnaire. Regression analysis was adopted. The researchers established that the respondents were well informed about the background and the purpose of this research and they were kept up-to-date with the participation process and regime. Every respondent was offered the opportunity to stay anonymous and their responses were treated confidentially. Consent was obtained from the appropriate authorities where copies of questionnaire were distributed.
